# Usefulness of Topically Applied Sensors to Assess the Quality of 18F-FDG Injections and Validation Against Dynamic Positron Emission Tomography (PET) Images

**DOI:** 10.3389/fmed.2018.00303

**Published:** 2018-11-01

**Authors:** Ronald K. Lattanze, Medhat M. Osman, Kelley A. Ryan, Sarah Frye, David W. Townsend

**Affiliations:** ^1^Lucerno Dynamics, LLC, Cary, NC, United States; ^2^Division of Nuclear Medicine, Department of Radiology, Saint Louis University, St. Louis, MO, United States; ^3^Doisy College of Health Sciences, Saint Louis University, St. Louis, MO, United States; ^4^A^*^STAR-NUS Clinical Imaging Research Centre (A^*^STAR), Singapore, Singapore

**Keywords:** infiltrations, extravasation, PET/CT, quality control, FDG

## Abstract

**Background:** Infiltrations of 18F-fluorodeoxyglucose (FDG) injections affect positron emission tomography/computed tomography (PET/CT) image quality and quantification. A device using scintillation sensors (Lucerno Dynamics, Cary, NC) provides dynamic measurements acquired during FDG uptake to identify and characterize radioactivity near the injection site prior to patient imaging. Our aim was to compare sensor measurements against dynamic PET image acquisition, our proposed reference in assessing injection quality during the uptake period.

**Methods:** Subjects undergoing routine FDG PET/CT imaging were eligible for this Institutional Review Board approved prospective study. After providing informed consent, subjects had sensors topically placed on their arms. FDG was injected into subjects' veins directly on the PET imaging table. Dynamic images of the injection site were acquired during 45 min of the uptake period. These dynamic image acquisitions and subjects' routine standard static images were evaluated by nuclear medicine physicians for abnormal FDG accumulation near the injection site. Sensor measurements were interpreted independently by Lucerno staff. Dynamic image acquisition interpretation results were compared to the sensor measurement interpretations and to static image interpretations.

**Results:** Twenty-four subjects were consented and enrolled. Data from 21 subjects were gathered. During dynamic image acquisition review, physicians interpreted 4 subjects with no FDG accumulation at the injection site, whereas 17 showed evidence of accumulation. In 10 of the 17 cases that showed FDG accumulation, the FDG presence at the injection site resolved completely during uptake corresponding to venous stasis, the temporary sequestration of blood from circulation. Static image interpretation agreed with dynamic images interpretation in 11/21 (52%) subjects. Sensor measurement interpretations agreed with the dynamic images interpretations in 18/21 (86%) subjects.

**Conclusions:** Sensor measurements can be an effective way to identify and characterize infiltrations and venous stasis. Comparable to an infiltration, venous stasis may produce spurious and clinically meaningful measurement bias and possibly even scan misinterpretation. Since the quality and quantification of PET/CT studies are of clinical importance, sensor measurements acquired during the FDG uptake may prove to be a useful quality control measure to reduce infiltration rates and potentially improve patient care.

**Registration:** Clinicaltrials.gov, Identifier: NCT03041090

## Introduction

In 2017, 90% of the ~3.1 million PET/CT studies performed in the United States were used to help oncologists diagnose, stage, plan treatments, assess tumor response, or longitudinally monitor cancer patients (1). It is expected that the number of PET/CT studies will continue to increase over time (1). Oncologic PET/CT studies require a prescribed dose of 18F-fluorodeoxyglucose (FDG) be injected as a bolus within 1 min, followed by a pre-defined uptake period (2). PET/CT scanner and procedural quality control (QC) help ensure the accuracy of the administered dose. For procedural QC, clocks are synchronized to ensure proper decay corrections, injection-to-image times are recorded to ensure longitudinal studies are comparable, and FDG delivery syringe residuals are measured (or estimated by some centers) and recorded to calculate actual administered dose (3–9). Currently, however, there are no routine QC measures to ensure that the entire administered dose actually enters the patient's vascular system.

An infiltration is the inadvertent paravenous administration of a solution or medicine into the soft tissue surrounding the vein (10). An FDG infiltration degrades PET/CT image quality and can reduce diagnostic sensitivity, when FDG is not delivered as a bolus and may therefore continuously enter the vascular system during the entire uptake period. Additionally, some infiltrations may cause artifacts that compromise the quality of the image (11). An FDG infiltration confounds quantification because the dose used in the standardized uptake value (SUV) calculation is not accurate. Based on the severity of the infiltrated dose, the effects to image quality and quantification may negatively affect patient care; for example, an ignored infiltration can result in mis-staging a patient's cancer, unnecessary and costly treatment, and understated SUVs (12, 13). A review of standard static images after the PET/CT procedure is one way to identify infiltrations, but the injection site may not always be in the imaging field of view (FOV) (14). When the injection site is in the imaging FOV, the severity of infiltrations can be misrepresented, since infiltrations can resolve during the uptake period and before imaging (15). Additionally, the effect of infiltrations on use of reference methods to correct SUVs is unknown.

Recognizing the importance of ensuring that the entire administered dose is properly injected into the venous system, a few centers have attempted to understand the magnitude of the infiltration issue by assessing static images of the injection site. Three centers in six studies involving 2,804 patients reported PET/CT infiltration rates ranging from 3 to 23% (mean 15.2%) (14, 16–20). These rates are likely underestimated due to the frequent exclusion of the injection site from the imaging FOV (14).

In the United States, ~12,500 PET/CT studies are conducted each workday (1). Estimating a 10% infiltration rate (<15.2%, the aggregate from the published rates previously cited) suggests that 1,250 patients may be infiltrated every day. However, not every infiltration is significant enough to negatively affect patient management. Furthermore, not every large infiltration happens in an imaging study that has patient management implications. Yet, the high infiltration rate suggests that a large number of patients may be negatively affected each year. Many interpreting physicians and most treating physicians who receive the scan reports will be unaware that these studies were compromised. Patients and their payers would also be unaware that their scans and results were negatively affected.

Currently available approaches to identify or assess the effect of infiltrations are not without limitations. Static imaging can sometimes capture infiltrations, but its lack of insight into the uptake period limits its use as an assessment tool for injection quality. Arterial blood sampling could provide an assessment of the injection quality but is invasive and unlikely to be routinely adopted in the clinic. Dynamic imaging of the injection site during the uptake period is likely considered the gold standard for assessing the quality of radiotracer injections; however, this approach may be impractical and negatively affects patient throughput in routine clinical static imaging protocols. While whole-body dynamic FDG PET imaging protocols may not always include the injection site in the imaging FOV, the impact of the infiltration would be addressed in the resulting quantitative measurements derived from these protocols. Existing whole body imaging protocols continue to be developed (21, 22) and may be introduced in the clinic in the future; however, these protocols may not always be logistically practical in certain clinical or high volume settings.

In a recent clinical study, investigative gamma scintillating sensors were applied to subjects with locally advanced breast cancer who were scheduled to undergo limited whole-body FDG-PET (15). Prior to injection of FDG, sensors were topically applied over the subject's palpable tumor, on their arms, and over their liver. Dynamic measurements in the form of a time-activity curve (TAC) were generated from tumor sensor data acquired during the uptake period and compared to tumor SUV. In several subjects, injection arm sensor measurements in the form of TACs detected and characterized radioactivity during the uptake period near the injection site. A review of the top-of-skull to toes image confirmed infiltrations were present at time of routine static imaging. Since the injection arm sensor measurements indicated the presence of radiotracer near the injection site the authors hypothesized that sensor measurements may prove a simple, practical, and useful way to provide QC to ensure the entire administered dose actually entered into circulation.

The aim of this study was to compare sensor measurements in the form of TACs with injection quality as assessed from dynamic PET imaging during the uptake period.

## Materials and methods

### Study design and participants

This study was carried out in accordance with the recommendations of the Belmont Report, the Declaration of Helsinki, the Nuremberg Code, and the St. Louis University Institutional Review Board. All subjects gave written informed consent in accordance with the Declaration of Helsinki.

Subjects referred to a PET/CT center for a standard-of-care FDG PET/CT examination were eligible to participate in this prospective, non-significant risk device study. The device (Lucerno Dynamics, Cary, NC) consisting of four sensors, a reader, adhesive pads, and software was used in the study to provide TACs. Subjects were enrolled in a convenience series for a 12-month period. Enrollment was dependent on staff and PET/CT scanner availability for dynamic image acquisition during the uptake period and simultaneous enrollment in a related study (17). Subjects under 18 years old, over 90 years old, or unwilling to tolerate four adhesive pads topically applied to their skin during the FDG uptake period were excluded from the study. Subjects also had to tolerate lying on the PET imaging table for 45 min of their 60-min uptake process. All subjects provided written informed consent.

### Test methods

Subjects followed standard clinical preparations for PET/CT imaging until technologists prepared to gain venous access. After evaluating potential venous access sites, subjects were positioned on the Philips Gemini TF 64-slice PET/CT imaging table so that the injection site would be at the caudal edge of the PET imaging bed. Technologists selected the location of the injection site and gained venous access according to their previous practice. This study did not impose any constraints on injection site selection, needle gauge, or type of venous access catheter or venipuncture needle used. After gaining venous access, technologists applied atraumatic adhesive pads to the subjects. One pad was placed ~7 cm proximal to the venous access site, the second mirrored on the contralateral arm, the third on the contralateral wrist, and the fourth over the liver. Sensors, connected to a reader that stored sensor output, were then attached to each pad.

Technologists could not effectively administer the FDG injection with the patient's injection site positioned in the eventual PET imaging bed. As a result, injections were administered on the imaging table outside the PET scanner and the sensors immediately began to record. After routine flushing of the venipuncture needle or catheter, technologists placed the subjects' arms alongside their torso and on plexiglass table extenders for support during the dynamic imaging acquisition process. Subjects were then advanced into the PET scanner to the pre-determined location for acquisition of dynamic images every 30 s for 90 frames. The time between injection and advancement into the PET scanner was recorded. Dynamic image acquisition ceased after 45 min, subjects were removed from the scanner, technologists stopped the reader, and sensors were removed. Subjects moved to an uptake room for the remainder of their uptake period and resumed the routine imaging protocol.

**Figure 1:**
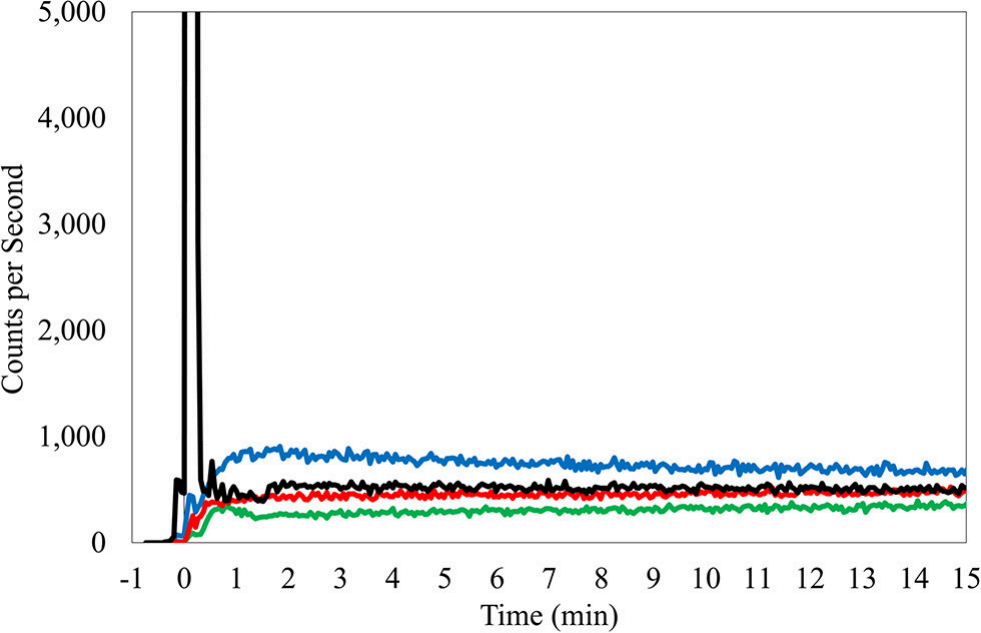
TAC from right antecubital fossa injection, butterfly access, 24 gauge needle. Black TAC, injection arm; Red TAC, reference arm; Green TAC, reference wrist; Blue TAC, Liver.

This PET center uses head-to-toe image acquisition as a standard imaging FOV for all cancer patients. Before or during the routine static imaging of the subject, technologists connected the Lucerno device reader to a PC. Data from the sensors were downloaded and basic information relevant to the injection procedure was recorded in the Lucerno software and on case report forms. Information included time between injection and first dynamic image acquisition, venous access site, venous access method, the name of the technologist who gained access and injected the subject, and amount of net administered dose. This information was then transferred via web interface to Lucerno for automatic processing and TAC generation (Figure 1). The resulting TACs were then made available to the technologists and Lucerno.

The Lucerno clinical staff interpreted the TACs. TAC interpretations were recorded in a data spreadsheet and explanations permanently logged with each TAC on the Lucerno viewing software. Dynamic image acquisitions and static images were reviewed by nuclear medicine physicians during their normal course of their daily practice at the center. Once the interpreting physician completed a subject's case report form with their interpretations, the technologist would gather these forms and add them to the technologist case forms for each subject. Periodically, several subjects' case report forms, including the physician's interpretations, would be faxed to Lucerno. The physician's interpretations would then be added to the respective subject's data spreadsheet. All sensor measurement and imaging interpretations were conducted independently.

Subjects did not receive additional radiation from participating in the study. Subjects were asked to complete a survey regarding use of the sensors after their entire imaging study was complete.

### Analysis

Two experienced and board-certified nuclear medicine physicians on staff at this center were available to interpret static images and dynamic image acquisitions. Physician interpretations looked for presence of radiotracer accumulation near the injection site. If presence was determined, further classification of minor, moderate, or significant presence of radiotracer was determined. The classification was qualitatively judged by the interpreting physician according to potential effect of detected radiotracer presence on SUV measurements; minor = not likely to have any effect, moderate = may have an effect, and significant = likely to have an effect on SUV calculation.

Dynamic imaging was chosen for the reference standard. Acquiring dynamic images every 30 s at the injection site allowed for nuclear medicine physicians to evaluate the presence of abnormal FDG accumulation throughout the uptake period with high sensitivity. Since static images are taken at this center ~70 min post-injection and since infiltrations are known to sometimes resolve during the uptake period (15), routine static imaging that included the injection site was not considered sensitive enough to qualify as the reference standard.

TACs were manually interpreted and classified based on learning developed at Lucerno. The manual interpretation process was initially developed by analyzing over 1,700 human, canine, and murine TACs from IRB and IACUC-approved studies over 5 years. The process was informed by reviews of literature, discussions with experienced and board-certified nuclear medicine physicians and physicists, and testing with phantom models. Additionally, TACs from ideal and known infiltrated human cases were studied to characterize injection quality reference (15). The process of manually classifying TACs was developed with assistance from nuclear medicine physician's interpretations of static PET images.

Manual interpretation of TACs considered several factors but is fundamentally based on an intuitive understanding and observations of ideal injection TACs. Ideal injections TACs are consistently similar in features. Reference arm counts remain low and injection arm counts peak immediately after administration, before rapidly declining to meet the reference arm levels (Figure 1). Interpretation of the TACs also considered center-specific policies and other considerations that can influence the shape of the sensor curves (e.g., centers that inject in the hot lab and then after 3 min, walk patients to their uptake rooms and patients who had their arms pressed close to the body, where torso radioactivity contribute to sensor counts). Interpretations included a review of the net administered dose, patient height and weight, and the injection site location. The interpretation also considered the side of the subject's body selected for the injection site with a review of the liver TAC and its potential influence on arm sensors. Interpretations also examined the slope of the bolus injection TAC as it approaches the reference sensor TAC, the absolute injection TAC counts at various points during the uptake period and the relationship to the reference arm counts at these same time periods, and the length of time that is required before the injection TAC approached within 50% of the reference sensor TAC. Additionally, area under the curve (AUC) ratios between injection and reference sensor TACs during various periods were compared (15). Furthermore, classification of the amount of radiotracer accumulated near the injection site, as reflected by the TAC, used standard infiltration terminology (none, minor, moderate, or significant amount of activity present).

Prior to presentation of interim data by a nuclear medicine physician from the center, Lucerno staff met with the center staff and reviewed the findings. The second review was of the complete data set. In subjects with partial data, no comparison could be made. During reviews, each subject's results were shared and discussed. TACs were presented and when needed dynamic image acquisitions and static images were reviewed.

Dynamic image interpretations of the subjects were compared to static image interpretations and to sensor TAC interpretations. True positives, false positives, false negatives, and true negatives were determined, and sensitivity, specificity, and accuracy were reported. Fisher's exact test of proportions was used to compare static image interpretation to sensor TAC interpretation.

The sample size was not determined in advance due to uncertainty of several factors: PET scanner availability, subjects willing to consent to lying flat on the PET imaging table for their uptake process, and sponsor funding.

## Results

### Presence of radiotracer

**Figure 2:**
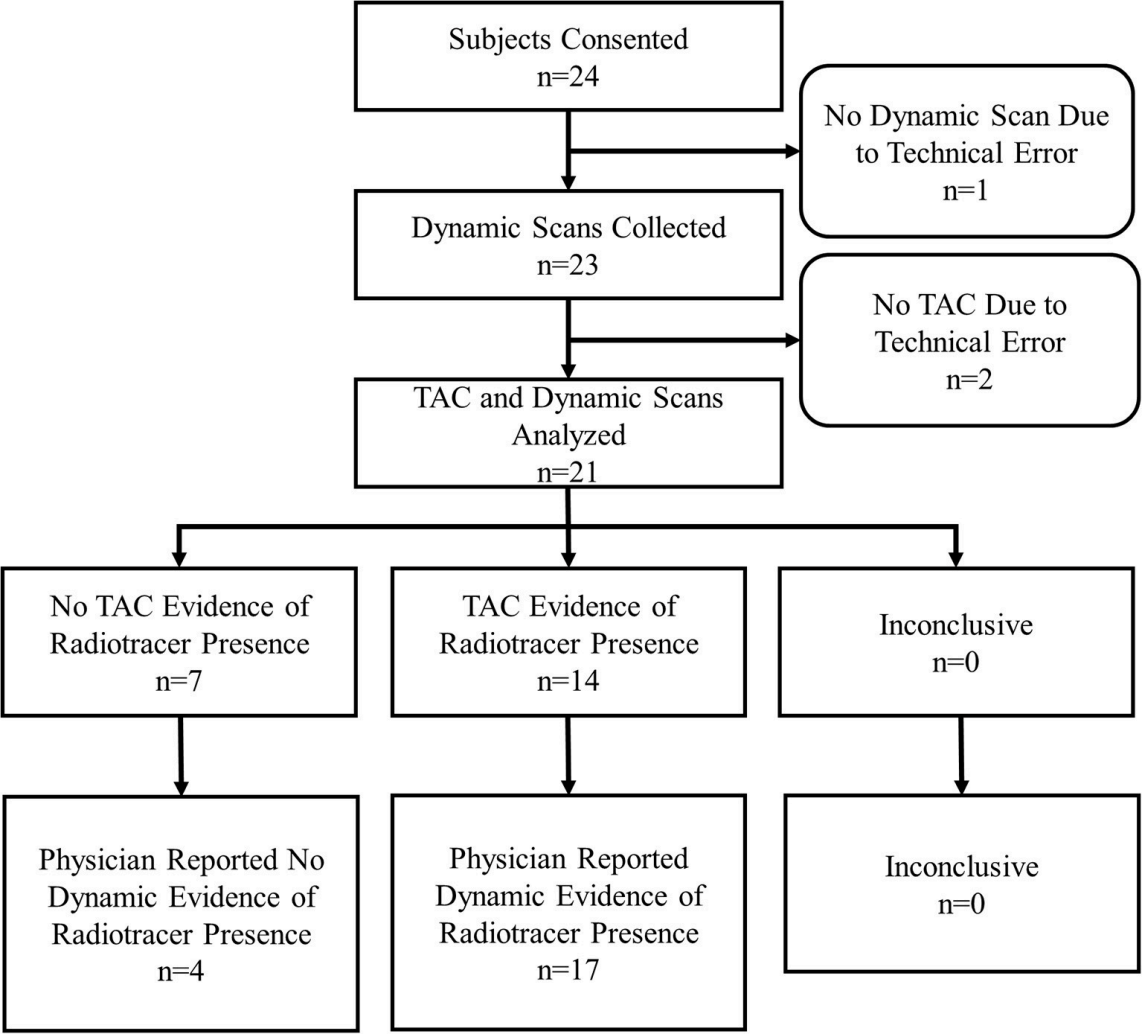
Subject flow chart.

During the 12 months of enrollment, 24 subjects consented to participate. Data from three subjects were not available for comparison. One subject had sensor data, but no dynamic images due to a malfunction of the PET imaging table that prevented the table from entering the PET scanner. As a result, no imaging was available for that subject. Two subjects underwent dynamic image acquisition, but had incomplete sensor data due to technologist error in starting TAC generation. This resulted in data collected for 21 evaluable subjects (Figure 2).

Of the 21 subjects analyzed, physicians reported dynamic image acquisition evidence of radiotracer presence near the injection site in 17 subjects and no evidence in 4 subjects (Table 1).

**Table 1:** Comparisons of static images and TACs interpretations vs. the reference standard, dynamic image acquisition interpretations.

**Subject**	**Dynamic reference standard**	**Static**	**TAC**	**Static vs. dynamic result**	**TAC vs. dynamic result**
1166	No evidence	No evidence	No evidence	True negative	True negative
1205	Infiltration	No evidence	Presence	False negative	True positive
1208	Infiltration	No evidence	Presence	False negative	True positive
1270	Infiltration	No evidence	Presence	False negative	True positive
1351	Infiltration	No evidence	Presence	False negative	True positive
1377	Infiltration	No evidence	Presence	False negative	True positive
1382	Infiltration	No evidence	Presence	False negative	True positive
1384	Infiltration	No evidence	Presence	False negative	True positive
1387	Infiltration	Infiltration	Presence	True Positive	True positive
1396	Infiltration	No evidence	No evidence	False negative	False negative
1398	Infiltration	No evidence	Presence	False negative	True positive
1399	Infiltration	Infiltration	No evidence	True positive	False negative
1464	Infiltration	Infiltration	Presence	True positive	True positive
1469	Infiltration	Infiltration	Presence	True positive	True positive
1578	Infiltration	Infiltration	Presence	True positive	True positive
1680	Infiltration	Infiltration	Presence	True positive	True positive
1714	No evidence	No evidence	No evidence	True negative	True negative
1894	Infiltration	Infiltration	Presence	True positive	True positive
1974	No evidence	No evidence	No evidence	True negative	True negative
1991	No evidence	No evidence	No evidence	True negative	True negative
2158	Infiltration	No evidence	No evidence	False negative	False negative

Physicians reported static image evidence of radiotracer presence near the injection site in 7 subjects and no evidence in 14 subjects. Physician reports of static images and dynamic image acquisitions were in agreement in 11/21 (52%) cases. In four of the 10 cases, where physicians' reports were not in agreement between static and dynamic, the dynamic acquisition images showed minor evidence of presence of radiotracer near the injection site and static images showed no presence. Comparing static and dynamic images (reference) resulted in 7 true positives, 0 false positives, 10 false negatives and 4 true negatives with sensitivity = 0.41, specificity = 1.00 and accuracy = 0.52.

Manual interpretation of sensor TACs reported evidence of radiotracer presence in 14 subjects and no evidence in 7 subjects. Manual interpretations of sensor TACs and physicians reports of dynamic images acquired were in agreement in 18/21 (86%) cases. Three cases, where physicians' reports and TACs were not in agreement were cases where evaluation of dynamic image acquisition reported evidence of minor presence of radiotracer and TAC evaluation found no presence. Comparing TACs vs. dynamic images (reference) resulted in 14 true positives, 0 false positives, 3 false negatives, and 4 true negatives with sensitivity = 0.82, specificity = 1.00, accuracy = 0.86.

Using dynamic image acquisition as the reference standard for injection quality, we compared the accuracy of TAC vs. static interpretations using Fisher's exact test of proportions. The *p*-value for testing the H0 of no difference in accuracy between TAC and static was 0.043, so we conclude that TACs were significantly more accurate than static images in assessing injection quality.

### Capture of traditional infiltration

**Figure 3:**
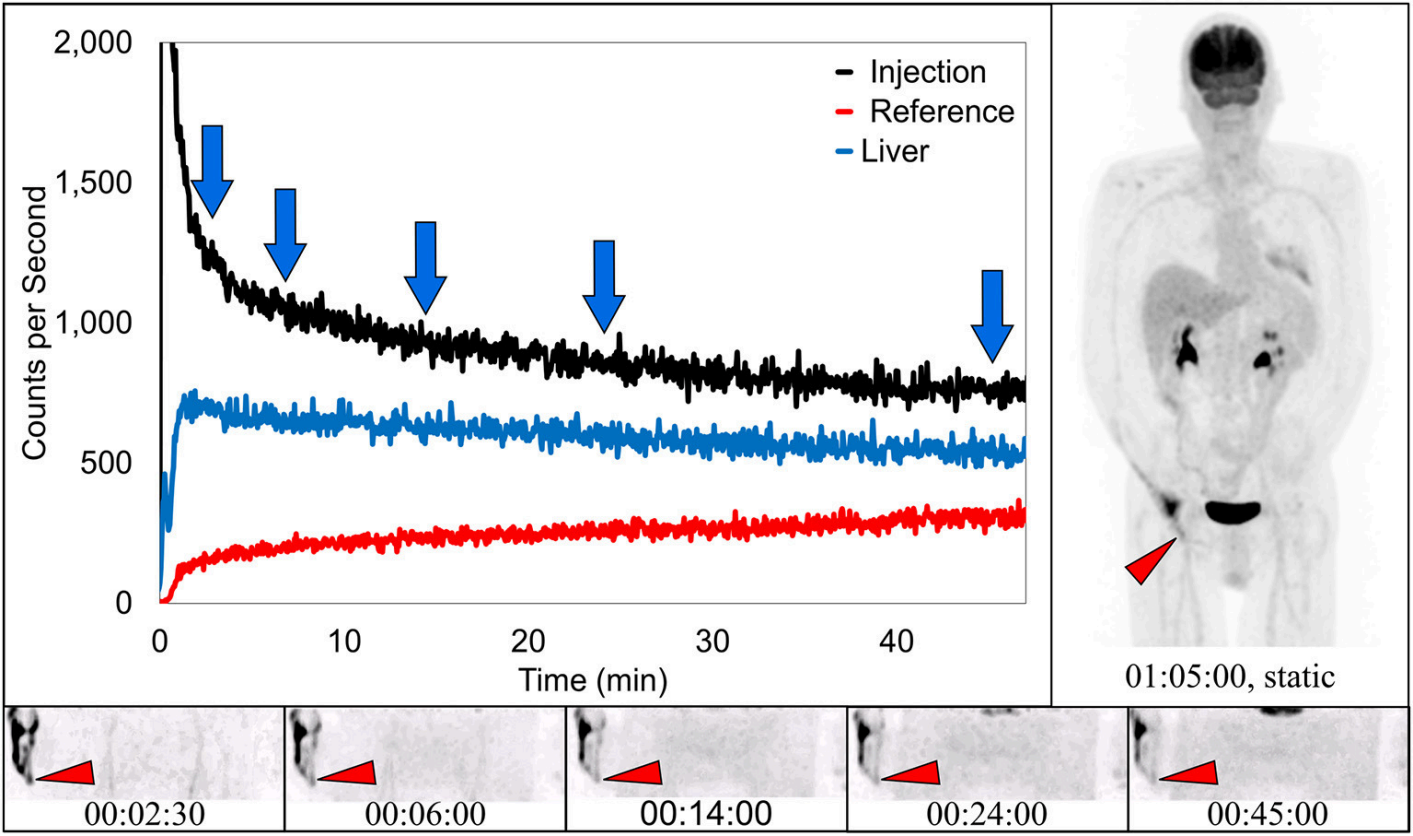
Dynamically acquired images (bottom row) taken at various times (blue arrows) during uptake, static image (upper right), and black TAC (top curve) from injection arm sensor, all reflect the resolving nature of an infiltration. Red TAC (bottom curve) reflects increasing uptake as captured by the reference sensor on the non-injection arm during the uptake period. Blue TAC reflects liver uptake and plays a negligible role in injection arm sensor TAC interpretation due to injection site location and nature of the liver uptake. Patient was positioned on PET imaging table to ensure the injection site would be located near the caudal edge of the PET imaging bed. Red arrows on dynamic image acquisition frames and static image indicate approximate injection site (right hand).

Dynamic image acquisition showed presence of radiotracer near the injection site in 17 subjects. In seven (41%) of these cases, static images taken ~70 min post-injection also showed evidence of presence of radiotracer, indicative of a traditional infiltration. One of these traditional infiltrations (TAC, several dynamic image acquisition frames, and static image) is shown (Figure 3). The paravenous nature of the injection is evident in these images.

### Capture of prolonged venous stasis

**Figure 4:**
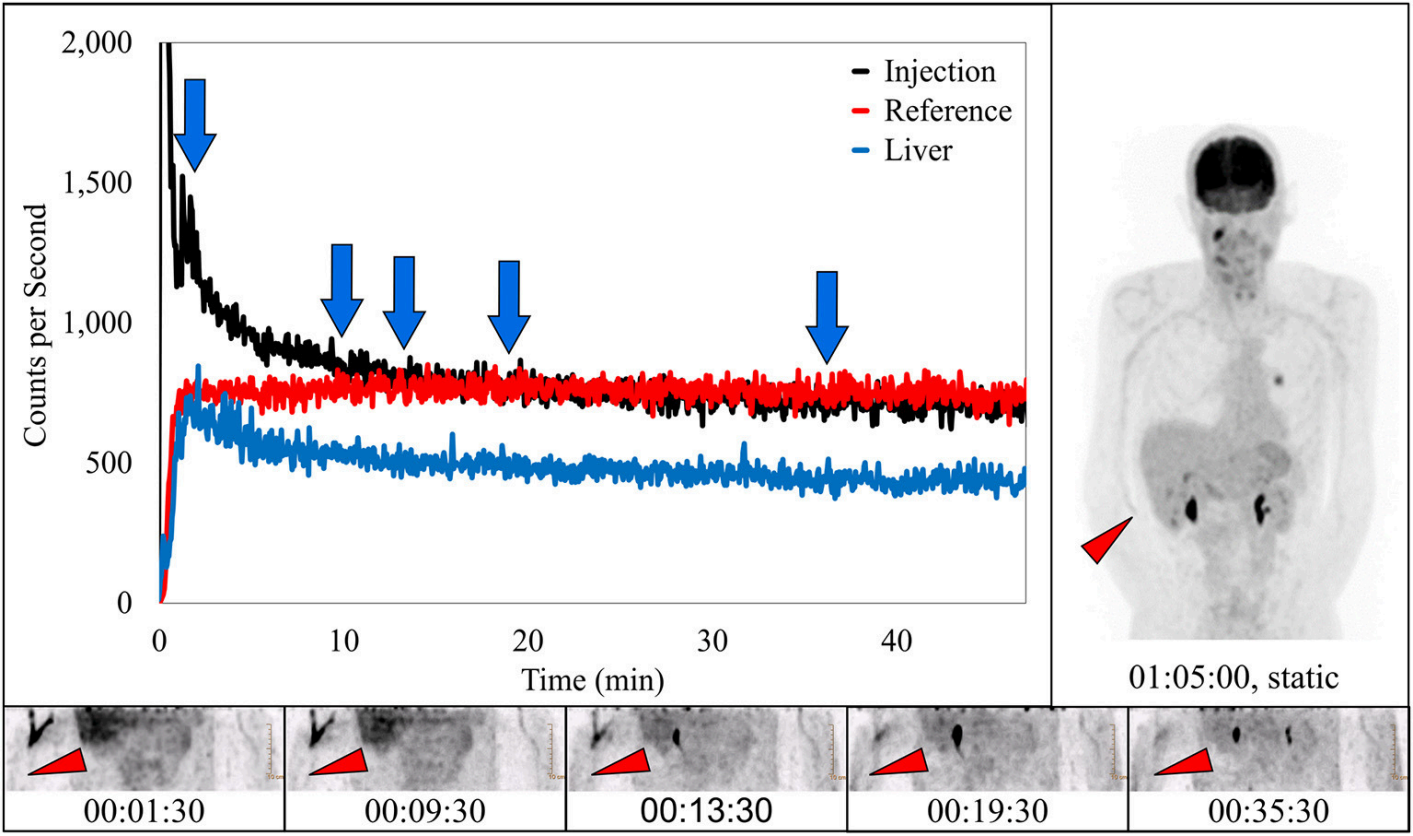
Dynamically acquired images (bottom row) taken at various times (blue arrows) during uptake, static image (upper right), and black TAC (top curve) from injection arm sensor all reflect the complete resolution of a prolonged venous stasis. Red TAC reflects uptake captured by reference arm sensor on the non-injection arm during the uptake period. Blue TAC reflects liver uptake. Proximity to injection arm sensor and nature of the liver uptake played some role in injection arm sensor TAC interpretation. Patient was positioned on PET imaging table to ensure the injection site would be located near the caudal edge of the PET imaging bed. Red arrows on dynamic image acquisition frames and static image indicate approximate injection site (right antecubital fossa).

In 10 subjects, dynamic image acquisition revealed prolonged radiotracer presence near the injection site during uptake that resolved completely prior to routine static imaging. While there was evidence in the dynamic image acquisitions, there was no indication of accumulation of radiotracer near the injection site in the standard static images. Dynamic image acquisition for these 10 subjects revealed a different presentation of the radiotracer; in these images, the radiotracer appeared contained within the venous system and resolved completely during the uptake period over different lengths of time (Figure 4). The resolution is also visible in the associated TAC. This phenomenon of the pooling of radiotracer within the venous system is similar to renal scan cases of prolonged venous stasis caused by venous obstructions (23).

### Capture of ideal injections

**Figure 5:**
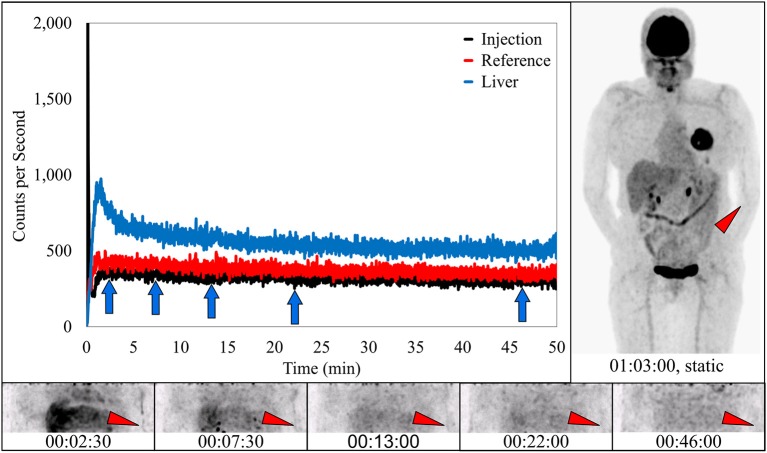
Dynamically acquired images (bottom row) taken at various time points (blue arrows) during uptake, static image (upper right), and black TAC from injection arm sensor all reflect an ideal radiotracer injection. Injection counts drop to very low levels immediately after bolus injection and saline flush, until the time the injection arm is placed in plexiglass table extenders and uncollimated sensors capture background torso radioactivity. At this time the injection curve climbs slightly and then levels off. Reference arm was placed in table extender before the injection. Red TAC reflects uptake captured by reference arm sensor on the non-injection arm during the uptake period. Blue TAC reflects liver uptake. Proximity to the reference arm sensor and nature of the liver uptake results in higher reference arm counts. Patient was positioned on PET imaging table to ensure the injection site would be located near the caudal edge of the PET imaging bed. Red arrows on dynamic image acquisition frames and static image indicate approximate injection site (left antecubital fossa).

In four subjects, dynamic image acquisition interpretation reported no evidence of infiltration. In these subjects the associated static images and sensor dynamic measurements interpretations were also indicative of ideal injections (Figure 5).

### Classification of radiotracer presence

In routine practice, guidelines suggest that physicians report infiltrations if these infiltrations are visible in the imaging FOV (24, 25). Physicians attempt to describe them as minor, moderate, or significant based on a qualitative review. The classifications are consistent with published guidelines, but the qualitative method does not provide a quantitative assessment of the amount of administered dose remaining at the injection site. Physician qualitative classification of static images, dynamic image acquisition, and Lucerno TAC classifications are reflected in Table 2.

**Table 2:** Characterization of radiotracer presence during dynamic image review captures presence of radiotracer at injection site.

**Classification**	**Dynamic images physician report of radiotracer presence at injection site**	**TAC Determination of presence of radiotracer at injection site**	**Static images physician report of radiotracer presence at injection site**
No Presence	4	7	14
Minor	9	13	6
Moderate	7	1	1
Significant	1	0	0

There were no adverse events reported during this study. On a scale of 0–10, where 0 represented no discomfort caused by sensors and pads, subjects rated the discomfort <1.

## Discussion

Dynamic imaging specifically of the injection site with quantification of radiotracer presence or the use of dynamic whole-body protocols would be the most effective way to assess the quality of radiotracer injections. However, our findings of 82% sensitivity and 100% specificity of sensor measurements for identifying the presence of radiotracer near the injection site suggest that sensor measurements may also be an effective radiotracer injection QC tool. While the use of topical sensors would not add incremental value in dynamic whole-body imaging scenarios, they could eliminate the need for specific injection site image acquisition during the patient's uptake phase. Our findings also suggest that TACs are more sensitive than static image review for identifying the presence of radiotracer, near the injection site, that is above and beyond what would be expected in the blood and tissue for two reasons. Injection sites may not always be in the routine static imaging FOV and static images cannot capture this excess radiotracer if it resolves before static images are acquired.

### Integration of sensors into current clinical practice

Use of the external sensors added ~30 s to the patient experience, 2 min to the technologist experience, and did not cause the patient any measurable discomfort. Results of the injection quality is almost immediately available to the center and the results can be interpreted on-site. This information may allow a clinician to determine, prior to imaging, whether exposing infiltrated patients to the additional radiation and time of the procedure is in the patient's best interest. In addition to providing individual QC information about each radiotracer injection, information provided along with sensor measurements can help centers actually reduce infiltrations (17). Because these injections are not routinely monitored in the US and because there is little immediate feedback to technologists, the rates are significantly higher than infiltration rates in other healthcare settings like chemotherapy and contrast CT injections (26–29). Reducing PET/CT infiltrations is important for baseline and subsequent scans. In a case of a single scan, an infiltration may impair the statistical quality of the image. In cases involving multiple scans, SUV comparisons could be incorrect due to an infiltration in one or both scans. In either of these scenarios, the quality of the injection process is critical to accurate interpretation of the scan and ensuring appropriate patient care.

### Potential short-comings and limitations

Table 2 suggests the limitations of qualitative assessments of visible infiltrations and sensor measurements in the classification of radiotracer presence and the need for more quantitative measures when infiltrations exist. In the one case where the physician classified radiotracer presence as significant during the qualitative review of dynamic images, manual TAC interpretation classified it as moderate. In the seven cases where the physician qualitative review of dynamic images classified radiotracer presence as moderate, manual TAC interpretation classified them as minor. In the nine cases where the physician qualitative review classified radiotracer presence as minor, manual TAC interpretation also classified six cases as minor and three cases as no presence of radiotracer.

Because quantifying the effect of the infiltration was not the intent of the study, the interpreting physicians did not quantitatively assess the radioactivity presence at the injection site in the dynamic image acquisitions or in the static images; nor did they gather normalization data or target region SUV data from the static images during the study. Having an estimate of the radioactivity may have resulted in a different outcome between dynamic image acquisition interpretation and sensor measurement interpretation. The estimation of the amount of radioactivity not available in circulation may also have provided valuable information regarding potential effect to the target region SUV. In addition, having an estimate and analyzing multiple SUV normalization methods may have provided insight into these methods.

Additionally, the design and use of only one sensor near the injection site creates other limitations. Both the unknown and potentially changing distance between the radioactivity and the sensor, as well as the role of the motion of the patient's arm with respect to other sources of radioactivity during the uptake period may also affect sensor measurements. While the design of the classification process and intended use of the device addresses many of the sensor limitations, it is possible the severity of infiltration or stasis could be misclassified from sensor measurement interpretation. Further efforts and studies are underway to provide estimates of the amount of radioactivity near the injection site.

When a PET/CT infiltration or stasis occurs, understanding the amount of radioactivity that was left near the injection site during the uptake period can highlight the potential effect of the infiltration and help physicians determine if the study should be repeated. Currently, the effects of infiltrations or stasis on scan interpretation are not yet understood. While it may be possible to correct for these administration issues through SUV normalization, the effects from an infiltration or stasis on SUV values of reference organs or the mediastinum blood pool are unknown (30). Therefore, further studies that assess the significance of the infiltration effects and how they correlate to current correction methods (e.g., normalization to blood pool or reference organ SUV) are needed.

We hypothesize that use of the plexiglass table extenders may have squeezed subjects' arms against their torso and this may have contributed to the high incidence of venous stasis in this subject population. The intent of the protocol was to attempt to simulate as much as possible the clinic's routine uptake process. As a result, the table extenders were used to ensure that the patients' arms were in the down position and not over their head during the uptake period. While the table extenders may have contributed to stasis in this study, we think the stasis topic may be a potentially important clinical issue. A venous stasis that resolves before imaging could be completely undetectable using standard static images, independent of the imaging FOV (Figure 4). We are currently evaluating the frequency of stasis encountered in standard protocols done without table extenders. While a venous stasis is not an injection infiltration, it may have the same potential effect on the quality and quantification of PET/CT images. And a stasis may be particularly confounding. If interpreting and treating physicians see a clear injection site or some minimal uptake contained in the vasculature in the image FOV, they may assume that the scan was produced with a high-quality injection. This misconception may negatively affect patient management. Further research is needed to evaluate the incidence and potential causes of venous stasis (e.g., rolling up sleeves for gaining venous access that produces a tourniquet-like effect) and whether these causes require procedural processes changes (23).

## Conclusion

Currently, there is no routine way to ensure the successful administration of a radiopharmaceutical into a patient's circulation. Since successful FDG administration is important to PET/CT image quality and SUV calculations, quality control is required. The results from this study suggest sensor TACs can be a patient-friendly QC measure to identify and characterize an infiltration or venous stasis near the injection site. TACs can provide additional insight regardless of imaging FOV or if infiltrations resolve prior to static imaging. Monitoring infiltration or stasis rates may prove valuable to improving the injection process and the quality and quantification of PET/CT studies. Alerting technologists and physicians to the fact that images have been negatively affected by an issue with FDG dose delivery may also be important to patient care.

## Author contributions

DT is responsible for protocol development and writing the manuscript. MO is the Principal Investigator responsible for protocol development, subject recruitment, data collection and analysis, writing and editing the manuscript. SF is the Research Coordinator responsible for subject recruitment, data collection and analysis, and editing the manuscript. RL is the Sponsor representative responsible for protocol development, data review and analysis, and writing and editing the manuscript. KR is the Sponsor representative responsible for data review and monitoring, and writing and editing the manuscript.

### Conflict of interest statement

RL and KR are employees of the sponsor. DT is a scientific advisor to the sponsor with no commercial or financial relationship. MO and SF received financial support for travel associated with delivery of preliminary results.
